# Trends in acute viral gastroenteritis among children aged ≤5 years through the national surveillance system in South Korea, 2013–2019

**DOI:** 10.1002/jmv.26685

**Published:** 2021-03-23

**Authors:** Seung‐Rye Cho, Su‐Jin Chae, Sunyoung Jung, Wooyoung Choi, Myung‐Guk Han, Cheon‐Kwon Yoo, Deog‐Yong Lee

**Affiliations:** ^1^ Division of Viral Diseases, Center for Laboratory Control of Infectious Diseases Korea Centers for Disease Control and Prevention Cheongju‐si Chungcheongbuk‐do Republic of Korea

**Keywords:** acute viral gastroenteritis, genotype, group A rotavirus, norovirus, seasonality, surveillance

## Abstract

Acute gastroenteritis is a global public health concern. This study aimed to analyze the trend and characteristics of acute viral gastroenteritis through a national surveillance network. Enteric viruses were detected in 9510 of 31,750 (30.1%) cases assessed from 2013 to 2019 by EnterNet. The most prevalent pathogens were norovirus (15.2%) and group A rotavirus (9.7%); most infections were reported in 2017 (34.0%). Norovirus and rotavirus coinfections were the most common. Norovirus infections were prevalent among 1‐year‐old children (1835 out of 9510 cases) during winter, and group A rotavirus infections were common during spring. Seasonality was not observed among enteric adenovirus, astrovirus, and sapovirus. The prevalent viral genotypes detected included norovirus GII.4, enteric adenovirus F41, astrovirus genotype 1, and sapovirus GI.1. However, changes in enteric virus trends were noted during the study period. Norovirus prevalence extended into spring, and new genotypes of enteric adenovirus, astrovirus, and sapovirus were identified. These surveillance data elucidate enteric virus epidemiological characteristics.

## 1 INTRODUCTION

Acute diarrhea disease is an infectious disease mainly caused by the consumption of contaminated water and food. It is known to occur due to complex factors such as public health and climate.^1^ According to the WHO report, 200 million cases of diarrheal diseases occur annually worldwide, and 1.9 million children under 5 years of age die each year from diarrhea in developing countries.^2^ Norovirus (NoV), group A rotavirus (RVA), enteric adenovirus (HAdV), astrovirus (HAstV), and sapovirus (SaV) are viruses associated with diarrheal disease.^3^ Among these, NoV causes outbreaks in people of all ages, is highly contagious, and spreads rapidly in crowded environments such as hospitals, cruise ships, playrooms, and residences.^4^ The pathogenic NoV genotype GII.4 causes large‐scale epidemics owing to the emergence of mutant strains and new genotypes.^5^, ^6^, ^7^, ^8^, ^9^, ^10^, ^11^ The prevalence of RVA infections has gradually decreased with the introduction of vaccines; however, the prevalence among infants and toddlers remains high.^12^, ^13^, ^14^ RVA genotypes are circulating at varying levels in different countries and regions.^13^, ^15^ Furthermore, various cases of HAdV, HAstV, and SaV infections have been reported at varying levels.^16^, ^17^, ^18^

Viral gastroenteritis is prevalent in winter with the cooler temperature and tends to decrease with an increase in temperature. The seasonality may vary depending on the region.^19^

In Korea, the nationwide acute diarrhea surveillance system (EnterNet‐Korea) has been operational since 2007. In total, 17 institutes of health and environmental research and 70 hospitals have collaborated to monitor the pathogens causing acute intestinal infections. The surveillance system curates and analyzes data online and shares information in real time. This study aimed to analyze the trends and characteristics of viral intestinal infections in Korea from surveillance data obtained from 2013 to 2019.

## 2 MATERIALS AND METHODS

### 2.1 Sample collection

Fecal samples were obtained from patients with symptoms of acute intestinal infection included in the nationwide surveillance system. Acute gastroenteritis was defined as primary symptoms including diarrhea, vomiting, abdominal pain, and nausea.

The survey was conducted for 7 years from 2013 to 2019, and fecal samples were collected from 70 hospitals nationwide from patients meeting the defined criteria for acute enteritis every week. Samples were transported to 17 institutes of health and environmental research immediately on collection or within a week and maintained at 4°C. Viral detection was carried out using the standardized surveillance protocols, and the results were submitted to the EnterNet system along with information regarding the sample.

### 2.2 Sample preparation

One gram of fecal sample was placed in 9 ml of sterile phosphate‐buffered saline (pH 7.4, Sigma) and sufficiently vortexed to prepare a 10% fecal suspension, which was then centrifuged at 4°C and 3000 rpm for 15 min to extract the supernatant containing the viruses. In the antigen detection test, the pretreated fecal supernatant was used immediately. For genetic analysis, nucleic acids were extracted from the pretreated solution using the commercially available nucleic acid extraction kit, that is, NucleoMag 96 virus kit (Macherey‐Nagel), in accordance with the manufacturer's instructions, using 200 µl of the pretreatment solution. The final nucleic acid extract was 50 µl and was stored at −70°C until subsequent use.

### 2.3 Virus detection test

For virus detection, antigen detection and gene detection were performed. RVA and HAdV antigens were detected using an enzymatic immunoassay. Pretreated 10% fecal supernatants were assessed using a RIDASCREEN EIA kit (R‐Biopharm). Gene detection was performed for NoV, HAstV, and SaV. Norovirus genotypes GI and GII were simultaneously detected using a commercially available real‐time reverse transcription PCR (RT‐PCR) assay, that is, AccuPower® Norovirus Real‐Time RT‐PCR Kit (Bioneer) and PowerCheck™ Norovirus GI/GII Multiplex Real‐time PCR Kit (Kogenebiotech). HAstV and SaV were detected through RT‐PCR analysis using specially generated primers (Table 1). RT was carried out at 48°C for 40 min, and gene amplification was performed through denaturation at 94°C for 15 min, followed by 35 cycles at 94°C for 30 s, 58°C for 30 s, and 72°C for 1 min, then approaching 72°C for 7 min for a final extension.

**Table 1:** Oligonucleotide sequences of primers used in this study

**Virus**	**Primer**	**Sequence (5ʹ–3**ʹ**)**	**Location**	**PCR product (bp)**	**Reference**
NoV GI	GI‐F1M (F)	ATG GCC ATG TTC CGI TGG ATG	5342–5671	314	In this study
	GI‐F2M (F)	CGG GCC CGA ATT YGT AAA TGA TG			
	GI‐R1M (R)	CCA ACC CAR CCA TTR TAC ATY TG			
NoV GII	GII‐F1M (F)	CCC TCG AGG GCG ATC GCA ATC T	5058–5401	313	In this study
	GII‐F2M (F)	CAC AAT TGT GAA TGA AGA TGG CGT CGA			
	GII‐R1M (R)	CCR CCI GCA TRI CCR TTR TAC AT			
HAdV	AD1 (F)	TTC CCC ATG GCI CAY AAC AC	1834–2296	482	Xu et al.^20^
	AD2 (R)	CCC TGG TAK CCR ATR TTG TA			
HAstV	mon269 (F)	CAA CTC AGG AAA CAG GGT GT	4526–4974	449	Noel et al.^21^
	mon270 (R)	TCA GAT GCA TTG TCA TTG GT			
SaV	SV‐F21 (F)	ANT AGT GTT TGA RAT GGA GGG	5157–5878	720	Okada et al.^22^
	SV‐R1 (R)	CWG GTG AMA CMC CAT TKT CCA T			

Abbreviations: HAdV, human enteric adenovirus; HAstV, human astrovirus; NoV, norovirus; RVA, group A rotavirus; SaV, sapovirus.

### 2.4 Genetic analysis

Genes were re‐amplified for positive samples. NoV was subjected to semi‐nested RT‐PCR (Table 1). During RT‐PCR analysis, RT was carried out at 47°C for 40 min, and denaturation was carried out at 94°C for 15 min, followed by 35 cycles at 94°C for 30 s, 54°C for 30 s, and 72°C for 45 s, then followed by a final extension at 72°C for 7 min. The product thus obtained was subjected to a second reaction cycle including denaturation at 94°C for 3 min, followed by 25 cycles at 94°C for 30 s, 56°C for 30 s, and 72°C for 45 s, then followed by a final extension at 72°C for 7 min. Amplification of HAdV was performed through PCR using a commercialized PCR kit (iMOD‐001TD, SNC) in accordance with the manufacturer's instructions (Table 1). The reaction conditions were denaturation at 94°C for 3 min, followed by 35 cycles at 94°C for 30 s, 50°C for 30 s, and 72°C for 1 min, then followed by a final extension at 72°C for 5 min. For HAstV and SaV, RT‐PCR products secured in the detection process were directly used for sequencing without additional experiments. The sequence was analyzed in a batch at the analysis center, and the results were submitted to the EnterNet surveillance system to confirm the genotype using the analysis tool in the EnterNet system. Sequences not analyzed using this analysis tool were analyzed using NCBI BLAST. RVA, whose genotype was not identified as an EnterNet system analysis tool, was excluded.

### 2.5 Data analysis

Among the data registered in the EnterNet system, the pathogen detection rate was analyzed using the data registered during the study period. Patients for whom data, including age or sample collection date, were not available were excluded from the study. Data were analyzed in accordance with the detection rate by pathogen, change in detection rate by period, age, mixed infection, and genotype. Age groups were categorized into 12‐month units: 0–11 months, age 0; 12–23 months, 1 year; 24–35 months, 2 years; 36–47 months, 3 years; 48–59 months, 4 years; and 60–71 months, 5 years. Data were analyzed using Microsoft Excel.

## 3 RESULTS

### 3.1 Sample information

According to the definition of acute gastroenteritis, 31,570 fecal samples were obtained from patients aged less than or equal to 5 years. Enteric viruses were detected in 9510 (30.1%) cases. NoV was the most frequently detected pathogen, found in 4802 (15.2%) cases, followed by RVA in 3056 (9.7%) cases. HAdV and HAstV were detected in 800 (2.5%) and 595 (1.9%) cases, respectively. SaV was detected in 257 (0.8%) cases. An annual average of 4510 samples was submitted. However, the system was reorganized after 2016 to secure 3000 samples per year. Owing to the reduction in the number of samples, only nationwide data, and not regional data, were analyzed (Table 2).

**Table 2:** Annual distribution of viral gastroenteritis in Korea, 2013–2019

	**No. of detection (%)**															
	**2013**		**2014**		**2015**		**2016**		**2017**		**2018**		**2019**		**Total**	
Pathogens	(*n* = 8018)		(*n *= 5915)		(*n *= 4173)		(*n *= 3360)		(*n *= 3757)		(*n *= 3520)		(*n *= 2827)		(*n *= 31,570)	
NoV	1064	(13.3)	954	(16.1)	621	(14.9)	556	(16.5)	659	(17.5)	414	(11.8)	534	(18.9)	4802	(15.2)
RVA	1118	(13.9)	561	(9.5)	406	(9.7)	213	(6.3)	374	(10.0)	248	(7.0)	136	(4.8)	3056	(9.7)
HAdV	132	(1.6)	186	(3.1)	61	(1.5)	142	(4.2)	102	(2.7)	134	(3.8)	43	(1.5)	800	(2.5)
HAstV	91	(1.1)	98	(1.7)	68	(1.6)	83	(2.5)	105	(2.8)	93	(2.6)	57	(2.0)	595	(1.9)
SaV	55	(0.7)	35	(0.6)	14	(0.3)	45	(1.3)	37	(1.0)	22	(0.6)	49	(1.7)	257	(0.8)
Total	2460	(30.7)	1834	(31.0)	1170	(28.0)	1039	(30.9)	1277	(34.0)	911	(25.9)	819	(29.0)	9510	(30.1)

Abbreviations: HAdV, human enteric adenovirus; HAstV, human astrovirus; NoV, norovirus; RVA, group A rotavirus; SaV, sapovirus.

### 3.2 Detection status by year

The average enteric viral detection rate was 30.1%, and except for 2017 (1277 cases, 34%) and 2018 (911 cases, 25.9%), the detection rate was similar. The average detection rate of NoV was 15.2% (±2.4); however, the rate varied from year to year. Except in 2013, NoV was the most frequent pathogen, and the pathogen detection rate was the lowest at 11.8% (414/3520 cases) in 2018. RVA continued to decrease until 2016 (6.3%, 213/3360) from a high detection rate of 13.9% (1118/8018) in 2013. The detection rate increased temporarily in 2017 (10%, 374/3757), but decreased again, being significantly lower in 2019 (4.8%, 136/2827). Similar to NoV, the detection rate of HAdV periodically increased and decreased. In 2014, 2016, and 2018, the HAdV detection rate increased to 3.1% (186/5915), 4.2% (142/3360), and 3.8% (134/3520), respectively. HAstV displayed a detection rate of less than 2% until 2015, but a detection rate of more than or equal to 2% from 2016. SaV displayed an average detection rate of less than 1% until 2016 where it was detected at 1.3% (45/3360) in 2016, 1.0% (37/3757) in 2017, and 1.7% (49/2827) in 2019 (Table 2).

### 3.3 Detection status by age

**Figure 1:**
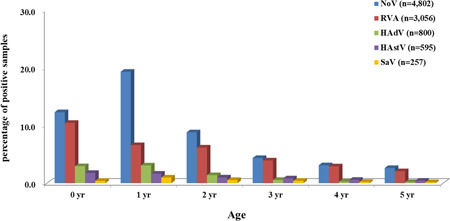
Age distribution of acute viral gastroenteritis cases in Korea, 2013–2019. In total, 31,570 samples were collected nationwide. The prevalence of viral diarrhea was the highest in the youngest age group (0–1 year). NoV was the most prevalent pathogen, followed by RVA, HAdV, HAstV, and SaV. Age group distributions were grouped in 5‐year segments: 0 month ≤ 0 year < 12 months, 12 months ≤ 1 year < 24 months, 24 months ≤ 2 years < 36 months, 36 months ≤ 3 years < 48 months, 48 months ≤ 4 years < 60 months, and 60 months ≤ 5 years < 72 months. HAdV, human enteric adenovirus; HAstV, human astrovirus; NoV, norovirus; RVA, group A rotavirus; SaV, sapovirus

Among children aged less than or equal to 5 years, the highest detection rate was 31.6% (3003/9510) at 1 year, followed by a high detection rate of 27.9% (2649/9510) at 0 years. The prevalence of NoV infections was the highest at 1 year of age at 19.3% (1835/9510); RVA, 0 years at 10.5% (994/9510). HAdV and SaV detection rates were as high as 3.1% (292/9510) and 1.0% (93/9510), respectively, at 1 year of age; HAstV, 1.8% (169/9510) at 0 years of age, similar to RVA (Figure 1).

### 3.4 Mixed infection

**Figure 2:**
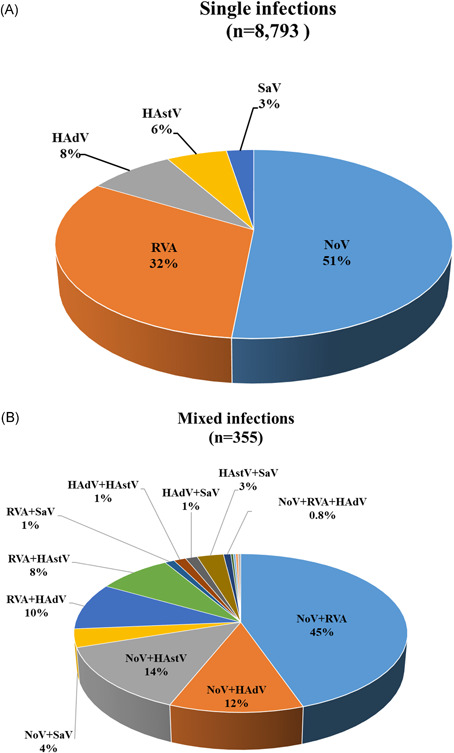
Distribution of cases of single (A) and mixed (B) enterovirus infections in Korea, 2013–2019. Distribution (%) of the cases of single (*n* = 8793) and mixed (*n* = 355) viral intestinal infections among 31,570 stool samples of children aged ≤ 5 years in Korea. The most frequent dual infections were NoV and RoV (45.9%, 163 of 355) infections. HAdV, human enteric adenovirus; HAstV, human astrovirus; NoV, norovirus; RVA, group A rotavirus; SaV, sapovirus

Among 9510 positive cases, single infections were detected in 8793 (92.5%) cases, and mixed infections were detected in 717 cases (8.2%). Among cases of single infections, NoV presented a high rate of 51.5% (4531/8793 cases); RVA, 32.1% (2823/8793 cases). Mixed infections were confirmed in 355 fecal samples, mostly involving two pathogens (348/355, 98.0%). NoV and RVA (163/355, 45.9%) were the most frequent, followed by NoV and HAstV (50/355, 14.1%). Furthermore, three viruses were detected simultaneously in seven fecal samples (Figure 2).

### 3.5 Seasonality

**Figure 3:**
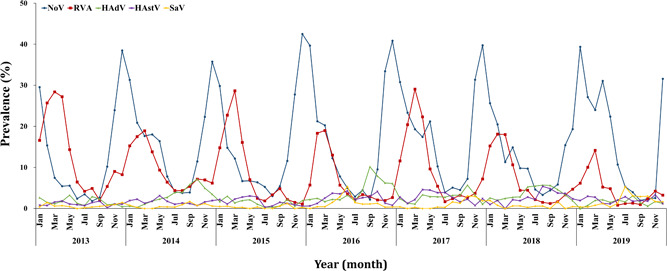
Seasonality of acute viral gastroenteritis infections in children aged ≤ 5 years in Korea, 2013–2019. The prevalence of acute viral gastroenteritis increased from October to April and then gradually decreased from May to September. NoV was predominant in winter (November–February), while RVA infections peaked in spring (January–May). HAdV, HAstV, and SaV displayed no clear seasonality. HAdV, human enteric adenovirus; HAstV, human astrovirus; NoV, norovirus; RVA, group A rotavirus; SaV, sapovirus

The enteric viral detection rate displayed typical seasonality. Detection rates peaked in January (48.9%, 1378/2819) and February (46.9%, 1318/2812), corresponding to winter in Korea. In summer, the lowest detection rate was recorded in July (12.1%, 326/2703). NoV, RVA, and HAdV displayed clear seasonality with relatively high detection rates. However, seasonality and periodicity were not observed for HAstV and SaV owing to the low detection rates of these viruses (Figure 3).

NoV presented a high detection rate between November and February, and the highest levels were in December (36.8% ± 7.9) and January (31.6% ± 5.3). However, in 2018, the detection rate increased from end‐November 2018 until early June 2019. RVA displayed a high detection rate between March and May during spring and then decreased during early summer. In particular, the RVA detection rate peaked in March (23.9% ± 6.2). HAdV displayed a relatively high detection rate during summer and autumn; from January to July, its average detection rate was less than 2.0%, but it was high in August (3.4% ± 1.7) and October (4.0% ± 3.0).

### 3.6 Genotype distribution

Genotypes were confirmed in 5508 samples of four viruses except for RVA (Table 3). NoV genotypes were analyzed in 4148 of 4802 (86.4%) positive cases: the GI group in 3.3% (136/4148) cases and the GII group in 94.6% (4012/4148) cases. The most prominent genotype in the GI group was GI.3 (28.7%, 39/136); GII group, GII.4 (55.6%, 2232/4012), GII.3 (14.3%, 574/4012), GII.2 (8.5%, 343/4012), and GII.6 (7.5%, 302/4012). The GII.17 type presented high detection rates from 2014 (5.1%) to 2015 (11.8%); GII.2 type, 2016 (7.2%) to 2017 (11.4%) and 2018 (21.6). The GII.3 genotype increased rapidly from 2013 (9.4%) to 2014 (16.6%) and from 2015 (14.3%) to 2016 (23.7%). The GII.4 genotype was significantly superior to the other genotypes by year.

**Table 3:** Annual genotypic distribution of viral gastroenteritis in Korea, 2013–2019

	Year	2013		2014		2015		2016		2017		2018		2019		Total	
Pathogens	Genotype	No.	(%)	No.	(%)	No.	(%)	No.	(%)	No.	(%)	No.	(%)	No.	(%)	No.	(%)
NoV	GI Group	27	(3.4)	36	(4.6)	17	(3.3)	11	(2.2)	10	(1.6)	27	(6.6)	8	(1.5)	136	(3.3)
	GII.2	34	(4.3)	15	(1.9)	13	(2.5)	36	(7.2)	72	(11.4)	88	(21.6)	85	(16.3)	343	(8.3)
	GII.3	74	(9.4)	131	(16.6)	74	(14.3)	118	(23.7)	86	(13.6)	38	(9.3)	53	(10.2)	574	(13.8)
	GII.4	439	(56.0)	412	(52.1)	279	(54.0)	249	(50.1)	380	(60.2)	170	(41.7)	303	(58.3)	2232	(53.8)
	GII.6	96	(12.2)	96	(12.1)	26	(5.0)	14	(2.8)	25	(4.0)	28	(6.9)	17	(3.3)	302	(7.3)
	GII.17	29	(3.7)	40	(5.1)	61	(11.8)	19	(3.8)	18	(2.9)	17	(4.2)	14	(2.7)	198	(4.8)
	Other GII Group	85	(10.8)	61	(7.7)	47	(9.1)	50	(10.1)	40	(6.3)	40	(9.8)	40	(7.7)	363	(8.8)
	Total	784	(100.0)	791	(100.0)	517	(100.0)	497	(100.0)	631	(100.0)	408	(100.0)	520	(100.0)	4148	(100.0)
HAdV	F40, F41	36	(64.3)	143	(98.6)	34	(97.1)	108	(86.4)	80	(82.5)	112	(87.5)	22	(68.8)	535	(86.6)
	Others	20	(35.7)	2	(1.4)	1	(2.9)	17	(13.6)	17	(17.5)	16	(12.5)	10	(31.3)	83	(13.4)
	Total	56	(100.0)	145	(100.0)	35	(100.0)	125	(100.0)	97	(100.0)	128	(100.0)	32	(100.0)	618	(100.0)
HAstV	HAstV 1	61	(95.3)	51	(64.6)	57	(91.9)	52	(70.3)	59	(60.8)	72	(79.1)	25	(45.5)	377	(72.2)
	HAstV 2	0	(0.0)	1	(1.3)	1	(1.6)	12	(16.2)	6	(6.2)	0	(0.0)	2	(3.6)	22	(4.2)
	HAstV 4	0	(0.0)	0	(0.0)	0	(0.0)	4	(5.4)	21	(21.6)	10	(11.0)	3	(5.5)	38	(7.3)
	HAstV 5	2	(3.1)	27	(34.2)	1	(1.6)	2	(2.7)	5	(5.2)	8	(8.8)	15	(27.3)	60	(11.5)
	HAstV 8	1	(1.6)	0	(0.0)	1	(1.6)	0	(0.0)	0	(0.0)	0	(0.0)	0	(0.0)	2	(0.4)
	Others	0	(0.0)	0	(0.0)	2	(3.2)	4	(5.4)	6	(6.2)	1	(1.1)	10	(18.2)	23	(4.4)
	Total	64	(100.0)	79	(100.0)	62	(100.0)	74	(100.0)	97	(100.0)	91	(100.0)	55	(100.0)	522	(100.0)
SaV	GI	29	(70.7)	22	(75.9)	9	(75.0)	30	(78.9)	32	(97.0)	19	(95.0)	34	(72.3)	175	(79.5)
	GII	9	(22.0)	5	(17.2)	2	(16.7)	0	(0.0)	1	(3.0)	1	(5.0)	6	(12.8)	24	(10.9)
	GV	3	(7.3)	1	(3.4)	0	(0.0)	3	(7.9)	0	(0.0)	0	(0.0)	0	(0.0)	7	(3.2)
	Others	0	(0.0)	1	(3.4)	1	(8.3)	5	(13.2)	0	(0.0)	0	(0.0)	7	(14.9)	14	(6.4)
	Total	41	(100.0)	29	(100.0)	12	(100.0)	38	(100.0)	33	(100.0)	20	(100.0)	47	(100.0)	220	(100.0)

Abbreviations: HAdV, human enteric adenovirus; HAstV, human astrovirus; NoV, norovirus; RVA, group A rotavirus; SaV, sapovirus.

HAdV genotypes were analyzed in 618 of 801 (77.2%) positive cases, revealing that F41 (86.1%, 532/618) was the most prevalent genotype, followed by A31 (0.9%, 6/618 cases) and F40 (0.5%, 3/618 cases). In 55 (8.9%) cases, B1.3, B1.7, C1, C2, C5, C6, and E4 were also identified to cause respiratory symptoms. In particular, in the past 3 years (2017–2019), in addition to the HAdV F41 genotype, fecal samples have revealed numerous genotypes of HAdV‐B and C types corresponding to the respiratory adenovirus.

HAstV genotypes were analyzed in 522 of 595 (87.7%) positive cases, revealing that Type 1 was the most prevalent genotype among 72.2% (377/522) cases, followed by Type 5 (11.5%, 60/522 cases) and Type 4 (7.3%, 38/522 cases). Various genotypes were detected in the past 3 years. In 2014, 27 (34.2%) cases of Type 5 infections were detected, and the detection rates for this genotype were high in 2018 (8.8%) and 2019 (27.3%). Type 4 genotypes were detected in 2017 (21.6%) and persisted in 2018 (11.0%) and 2019 (5.5%) as well.

SaV genotypes were analyzed in 220 of 257 (85.6%) positive cases, revealing the GI group in 79.5% (175/220) cases, GII group in 10.9% (24/220) cases, and the GV group in 3.2% (7/220) cases. In the GI group, the GI.1 genotype revealed the highest detection rate of 60.0% (132/220 cases).

## 4 DISCUSSION

Viral acute gastroenteritis is a prominent disease in children.^10^, ^16^, ^17^, ^18^ NoV was the primary cause of acute gastroenteritis in Korea during the study period. The NoV detection rate was similar every year; however, the genotype displayed various epidemic patterns. Since the mid‐1990s, GII.4 has been the primary genotype, and new variant strains have emerged every 2–3 years.^7^, ^23^ GII.17 and GII.2 are the major NoV genotypes causing infections in northeast Asian countries, including Korea, China, and Japan.^8^, ^9^, ^10^, ^11^ While GII.4 remains an important genotype, GII.17 and GII.3 types were prevalent in 2014–2015 and GII.2 and GII.3 in 2016–2017. In 2018–2019, the GII.2 genotype became more widespread; however, the trend changed to the GII.4 type, and the NoV epidemic continued until spring. Recently, the new genotypes GII.17, GII.2, and GII.3 have tended to be prominent contributors to the epidemic. The GII.4 genotype cannot generate an effective mutant strain to lead the epidemic. Nevertheless, the pathogenicity of the GII.4 genotype may be greater than that of the other NoV genotypes. Furthermore, studies on NoV have exclusively focused on the antigenicity of the virus. However, recent outbreak analysis has confirmed that NoV RNA‐dependent RNA polymerase (RdRp) is rearranged with other genotypes.^10^, ^11^, ^24^ Therefore, CDC and Noronet have been analyzing RdRp regions, along with the existing capsid sites, to identify new NoV recombinants or strains. Therefore, to clearly understand the long‐term prevalence and mutation patterns of NoV, additional surveillance of RdRp is required, along with antigenic changes in the existing capsid site.

Rotavirus vaccine has been introduced in Korea and infants and toddlers are becoming exposed to it. Since this surveillance project is not a prospective cohort study on vaccination, the direct effect of vaccines cannot be determined, but it was confirmed that the rate of pathogen detection had continuously decreased. As the detection period of norovirus has recently extended until summer, it was expected that the detection rate of rotavirus would also be delayed, but the correlation between the two pathogens was not confirmed. However, the yearly decrease in the detection rate of rotavirus may be due to vaccination, but it seems to be related to the report that the outbreak of rotavirus is delayed and moving.^13^, ^14^, ^15^, ^25^, ^26^

Around this time, enteric HAdV, HAstV, and SaV tend to have newer genotypes relative to the previous ones. The detection rate of HAdV B and C genotypes was increased in addition to that of the F genotype, which was the predominant genotype, along with the detection rate of HAstV Type 4 and Type 5. Furthermore, the emergence of new genotypes is associated with the prevalence of new pathogens.^27^, ^28^, ^29^

The present surveillance data potentially broaden the current understanding of the epidemiological characteristics of enteritis‐causing viruses. However, the surveillance data could not account for changes in the prevalence of NoV and new genotypes. The present data would help understand this epidemic by further analyzing the environmental factors associated with this disease, such as its correlation with weather‐related factors and the genotype epidemic period, along with continuous monitoring of the pathogen detection rate.

## AUTHOR CONTRIBUTIONS

*Writing‐original drafting*: Seung‐Rye Cho. *Formal analysis and methodology*: Su‐Jin Chae and Sunyoung Jung. *Writing‐review and editing*: Wooyoung Choi and Myung‐Guk Han. *Obtained funding*: Cheon‐Kwon Yoo. *Conceptualization and supervision*: Deog‐Yong

## Data Availability

Data available on request from the authors.
